# Analysis of the Effects of Rubber Dosage and Digestion Time on the Mechanical Properties of Low Dosage Crumb-Rubber-Modified Asphalt Concrete Mixtures

**DOI:** 10.3390/ma18071419

**Published:** 2025-03-23

**Authors:** Greg White, Andrew Kidd

**Affiliations:** 1 School of Science, Technology and Engineering, University of Sunshine Coast, Sippy Downs, QLD 4556, Australia; 2 Brisbane City Council, Brisbane, QLD 4001, Australia; andy.kidd@brisbane.qld.gov.au

**Keywords:** crumb rubber, modification, binder, asphalt, mechanical properties

## Abstract

Crumb rubber modification of bituminous binders for asphalt concrete mixture production has been shown to provide significant environmental benefits, in terms of reduced embodied carbon, as well as improvement in the mechanical performance properties of asphalt mixtures. Furthermore, even at low dosages of crumb rubber, significant anti-ageing benefits have been reported, in terms of oxidation and ultra-violet light exposure. However, the effect of low dosage crumb rubber modification on the mechanical properties of asphalt mixtures must be understood. This research compared otherwise nominally identical dense-graded asphalt mixtures produced with crumb rubber modified binder at 5%, 10%, and 15% (by weight of the bitumen) and, using short digestion (reflecting field blending) and long digestion (reflecting terminal blending), to two control asphalt mixtures across a range of mechanical properties indicative of stiffness, rutting resistance, fatigue cracking resistance, cold fracture resistance, and moisture damage resistance. It was concluded that 10% was the optimum crumb rubber content and that crumb rubber modification generally improved the mechanical properties of asphalt mixtures, particularly the deformation resistance and the fatigue cracking resistance, which were both improved significantly. However, the effect of crumb rubber content and digestion times was variable. Consequently, the decision to field blend (short duration) or terminal blend (long duration) should be based on logistics, and not on asphalt mechanical properties and the associated mixture performance.

## 1. Introduction

All asphalt mixtures are a combination of aggregate (crushed quarried rock), bituminous binder (from the distillation of crude oil), and inert or chemical fillers (such as crusher dust, lime or cement) [1]. Air voids are also incorporated to stop the bituminous binder displacing the aggregate particles, thereby inhibiting the physical interlock and friction between the particles, resulting in mixture instability [2]. Although asphalt mixtures are often designed to maximize their structural performance, their durability is also important, as this significantly effects the whole of life proposition of the surface, and therefore the pavement [3].

Sustainability is arguably the most important new topic for pavement engineering practitioners and researchers in the 21st century [3]. There are many opportunities for achieving more sustainable asphalt surfaces for flexible pavement construction, including the incorporation of recycled asphalt, processes crushed waste glass, processed waste plastic, and crumbed rubber from used vehicle tires [4]. It has been shown that of all these opportunities, materials that partially or completely replace the bituminous binder are likely to provide the greatest embodied carbon and financial cost savings, particularly when the bituminous binder is polymer-modified [5]. This reflects the high financial cost and the high embodied carbon in the both the bitumen and the polymer. Despite the reuse or recycling of the waste and byproducts into asphalt mixtures gaining significant attention in recent years, it is critical that the performance of the asphalt mixture, and its expected service life, are not comprised. That is because even a one- or two-year reduction in the service life of an asphalt surface is likely to reverse any potential benefit achieved by using a waste material or byproduct [6].

Recycled asphalt (RA) is arguably the most established and common material to be incorporated into more sustainable asphalt mixtures and has been shown to provide the greatest financial cost and embodied carbon savings in certain circumstances [7]. However, the use of crumb rubber (CR) to extend and modify the bituminous binder has also seen increased interest in recent years [4,8]. This is likely to reflect the potential to improve the performance of the binder and the asphalt mixture in a similar manner to synthetic polymers, for example, styrene–butadiene–styrene, as well as the consumption of existing stockpiles of used vehicle tires for the production of the CR [9,10].

Although CR can be incorporated using a dry mixing approach, where it is added directly to the asphalt production plant, it is more common to wet blend it into the bitumen [11,12,13]. As explained below, this can be performed in the field or at a bitumen refining or modification terminal [14,15]. The main difference between the two methods is the time allowed for the digestion of the crumb rubber into the bitumen phase [16,17]. Furthermore, the CR can be added at a range of contents, usually expressed as a percentage of the mass of the bitumen. Most research on crumb rubber modification (CRM) of bituminous binders and asphalt mixtures has focused on higher dosages of CR, typically 20% to 30% (by mass of the bitumen), for improvement in the mechanical asphalt mixture properties, similar to that provided by conventional polymers [18]. However, the same research has also reported significant photo-oxidative ageing performance improvement associated with CRM of asphalt mixtures [19,20,21,22,23,24,25]. This has motivated researchers to consider the effect of lower dosages of CR, typically 5% to 15%, for cost-effective ageing improvement of asphalt mixtures for use in local roads, where expensive conventional polymer modification would not normally be required [14,26]. Although the focus on this research has been on the anti-ageing effects of CRM, the effect of the CR on the mechanical performance of the asphalt mixtures is also important [27,28,29,30].

The aim of this study was to analyze the effects of both CR dosage and CR digestion time on the mechanical or physical properties of asphalt mixtures for local road surfacing, when modified with relatively low dosages of crumb rubber. Eight otherwise nominally identical asphalt mixtures were produced, including six CRM binders and two control binders. Laboratory testing was performed on each, for properties indicative of stiffness, deformation resistance, fatigue and fracture resistance, in addition to moisture damage resistance. The results were statistically analysis to determine the effects of both CR content in the binder, and the time for CR digestion into the binder. It is expected that the results of this study will support the development and use of CR-modified asphalt mixture for the surfacing of local road pavements.

## 2. Background

### 2.1. Asphalt Binder and Mixture Ageing

Bitumen ageing significantly affects bituminous binder and asphalt mixture properties [31,32,33]. For example, softening point and flash point temperatures increase with ageing, while penetration and ductility reduce as the result of ageing [34]. In terms of asphalt mixtures, ageing increases the Marshall stability and decreases the Marshall flow [35]. The stiffness modulus of aged asphalt samples also increases by up to three times, compared to original modulus values [35].

It is also well established that binder and mixture ageing significantly affects ravelling in asphalt mixtures, particularly for mixtures that have an unusually high air void content, such as open-graded asphalt mixtures [36]. However, ageing is also important for airport pavements [31] and local roads, because resurfacing of these pavements is generally triggered by shards or spalls of the asphalt, generated by fretting and ravelling of the surface, presenting a safety issue for aircraft engines and undercarriages [37].

With the intent of extending the service life of pavement surfaces due to age-related distress, researchers have considered methods to slow or reverse asphalt binder and mixture ageing [31]. Warm mixed asphalt technology is one option to reduce oxidation of the binder during asphalt production [38]. For mixtures that contain significant portions of RA, rejuvenators are commonly added to offset the effect of the more aged binder from the RA [39]. Some researchers have also reported reduced in-service binder ageing associated with the use of polymer modification of the binder [40] and even for use of recycled plastic modified binders [41]. Once the surface is constructed, preservation treatments and products have been used over the years, intended to slow or even reverse the effect of binder and mixture ageing, particularly for local roads [42] and for airports [43]. Crumb rubber has been found to have the same effect on ageing of asphalt binders and mixtures as synthesized polymers do [44].

### 2.2. Crumb Rubber as an Asphalt Binder Modifier

As mentioned above, crumbed tire rubber is an established technology for the use of an otherwise waste material for the improvement of the performance of asphalt mixtures [4]. For example, CRM of PG 64-22 unmodified binder was found to produce asphalt mixtures with deformation resistance, cracking resistance and elastic modulus values that exceeded otherwise identical asphalt mixture produced with PG 76-22 polymer-modified binder [45]. However, despite these positive laboratory results, field trials have produced variable results and highlight the complexity of CRM binders [46,47,48].

There is the source and size of the rubber crumbs. Smaller crumbs produce a more homogenous binder, but the cost of processing is higher [49]. The more finely graded CR products, with associated increases in surface area, result in faster and more complete reactions during blending [50,51,52], which are largely a function of maltene penetration ability and rates and are inversely related to particle size [9]. Furthermore, the method of blending can be wet or dry. Dry mixing is where the rubber is added directly to the asphalt production plant with the aggregate, while wet mixing sees the crumb rubber pre-blended into the bitumen prior to asphalt production [53,54]. Wet blending produces a more homogenous asphalt mixture and increases dissolution into the bitumen [55], but the crumbed rubber can segregate out during storage and transportation [56]. Also, for wet-mixed CRM, the mixing can be performed at a bitumen refinery (known as terminal blending) or in a mobile facility on site (known as field blending) which affects the time between blending and asphalt production, which in turn affects the degree of dissolution of the CR into the bitumen phase [54,57].

The issues mentioned above relate largely to the degree of dissolution of the CR particles into the bituminous binder phase, as a result of the post-blending storage time. Unlike conventional polymers, crumbed rubber does not completely dissolve when mixed into bitumen [58,59,60]. Rather, a multi-phase product consisting of solid rubber particles and bitumen containing dissolved rubber is produced [56]. At the interface between the solid rubber and the solid bitumen is a gel phase consisting of semi-liquid and semi-solid rubber [61]. The relative volume of each phase of CRM binder depends on the rubber type, particle size, bitumen–rubber compatibility, and the time and temperature of mixing [62,63,64]. Furthermore, it is not clear how the different phases of crumb rubber should be considered in a volumetric-based asphalt mixture design method [65,66]. This creates a challenge when attempting to isolate the effect of the CR on asphalt mixture properties, which require the preparation of samples of otherwise identical samples, including the bituminous binder content.

### 2.3. Mechanical Properties of Asphalt

The mechanical properties of asphalt mixtures for pavement surfacing are as follows:

- Stiffness;
- Deformation resistance;
- Fatigue resistance;
- Fracture resistance;
- Moisture damage resistance.

Asphalt mixture stiffness indicates the contribution of the asphalt to the structural capacity of the pavement [67,68]. Stiffness is characterized by an elastic modulus and can be measured in the laboratory in indirect tension, bending (flexure), or compression [1]. Resilient modulus, measured on cylindrical specimens under cyclic indirect tensile loading, is a common measure of asphalt mixture stiffness [69].

Resistance to deformation includes resistance to plastic flow, as well as post-construction densification [70]. Good resistance to deformation is important to avoid asphalt rutting, shoving, and shearing, and it increases pavement smoothness and prevents ponding of water from inhibiting tire skid resistance. The rut depth of slabs or beams after 10,000 or more load cycles of a wheel tracking device is a common indicate of deformation resistance [69].

Resistance to fatigue is focused on cracking due to repeated loading at the intermediate or working temperature range [71]. Many asphalt fatigue tests are available, including beams in repeated three-point bending, beams in repeated four-point bending, cylindrical specimens in repeated indirect tensile loading, as well as various specimens in direct tension [1]. The four-point bending test, based on the calculated reduction in stiffness or modulus to indicate the end of the fatigue life, is common [69].

In contrast to fatigue resistance, fracture resistance is more associated with brittle fracture at low temperature [72,73]. The indirect tensile asphalt cracking test, known as IDEAL-CT, was developed as a simple, low-cost, and repeatable method for determination of fracture properties [74]. Where monotonic loading is applied to cylindrical samples, the resulting load–displacement curve is analyzed using fracture mechanics to determine an index of energy required to cause brittle fracture [75,76].

Moisture resistance is the ability of the asphalt to retain its integrity in the presence of moisture and is an indicator of the ability to avoid binder stripping over a long service life [77]. Stripping resistance is complex, but there are well-established test methods for the measurement of the resistance to stripping of asphalt mixtures [78], with the tensile strength ratio (TSR) test being a common method, which is also known as the Lottman test [79].

Other properties of interest for asphalt mixture performance also include fretting and ravelling resistance and workability. Fretting and ravelling resistance are particularly important at the bituminous binder and asphalt mixture age [31] and there is not yet an agreed test method for measuring age-related fretting and ravelling resistance [31]. Workability is the ease with which an asphalt mixture can be paved and compacted in the field [80]. However, this is not an issue for low dosage CR-modified binders and asphalt mixture, which have been demonstrated to be at least as workable as polymer-modified asphalt binders and mixtures [5].

## 3. Methods and Results

Eight bulk samples of 10 mm-sized dense-graded asphalt were produced in the laboratory. This mixture was designed as a surfacing mixture for local road pavements. One sample was produced with an unmodified binder, known as C170, and one with an acid-modified binder that is commonly used in the local area, known as ‘multi-grade’ or M1000 [81]. The other samples were prepared with a binder modified with crumb rubber, at dosages of 5%, 10%, and 15%. At each CR content, long-duration blending (representing terminal blending) and short-duration blending (representing field blending) were used. Each bulk sample was sub-sampled, and specimens were prepared and tested for various performance properties, including stiffness, fatigue resistance, fracture resistance, rutting resistance, and moisture damage resistance. The results were compared graphically and by using simple statistics, with Student’s t-test *p*-values used to determine the statistical significance of differences between mixture properties for samples with replicate measurements. Student’s t-test is appropriate because of the small sample size of otherwise normally distributed results [82]. A *p*-value of 0.05 or less was considered to indicate that the property was significantly different for asphalt mixtures with different binders.

### 3.1. Materials

**Figure 1:**
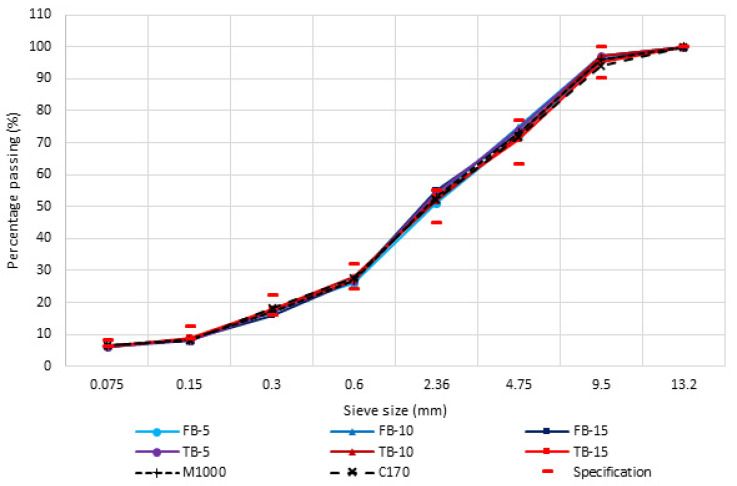
Bulk asphalt sample aggregate gradations.

All bulk asphalt samples were prepared according to the Brisbane City Council 10 mm dense-graded Type 2 requirements. The aggregate gradation of each bulk sample was similar (Figure 1), ensuring that the effect of the CR-modified binder was isolated. The control mixture contained 5.9% (by mass) unmodified C170 bitumen. The modified mixtures contained 5%, 10%, and 15% (by mass of the bitumen) long- and short-duration blended CR sourced from a commercial supplier. To provide asphalt mixture samples with the same target air void content, the CRM binder content was increased to account for the CR (Table 1), as determined previously [65]. The properties of the eight binders are compared in Table 2.

The bulk sample designations and characterizations are summarized in Table 3. The similarity in the aggregate gradations means that the differences in the asphalt mixture properties were isolated to the differences in the binder properties. It is clear from the results in Table 2 that the CR increased the elasticity of the binders, indicated by the torsional recovery results increasing from 4% (C170) and 14% (M1000) up to 14% to 41% (short-blended) and 14% to 28% (long-blended). Although the penetration was not significantly increased compared to the unmodified bitumen (C170), the relatively low penetration of the acid-modified M1000 was clear. This low penetration, high viscosity, and plastomeric nature of M1000 has been linked to premature and age-related cracking of asphalt surfaces [83], so the higher penetration for the CR-modified binders is preferable. As the CR content increased, the CR-modified binder viscosity increased significantly, but the blending duration did not significantly affect this increase in viscosity. Similarly to polymer-modified binders, the CR-modified binders were susceptible to segregation, particularly at 15% CR content, and with long-blending durations. This was likely attributable to the loss of the natural and synthetic rubber fractions from reaction and dissolution effects, where the associated reduction in swelling and rubber particle size, combined with a relative increase in the carbon black and filler contents, significantly increased the remaining CR particle density, resulting in the settling of CR particles from suspension in the binder phase [84]. Although the samples used in this research were not stored long enough for segregation of the rubber to be a potential issue, this issue must be managed for routine use in asphalt production.

The CRM binders were prepared using a truck tire-derived, 30 mesh CR product meeting the relevant Australian specification requirements, with 100% passing the 1.18 mm sieve. The same C170 feedstock was modified by following a standard laboratory procedure [84]. The CR was incorporated into the C170 bitumen using a Silverson high-shear mixer. For the short-blended products, blending was conducted at a reaction temperature of 190 °C and a mixing speed of 300 rpm for 5 h. This method was adopted to allow for adequate reaction time without excessive dissolution of the CR particles from either high temperature or high-shear effects and was intended to reflect field blended products. Long-duration blending samples were initially prepared the same as the short-blend products but were then further heating to a temperature of 180 °C, without further shearing, for a total of 48 h. This was intended to represent products produced and stored at terminal facilities prior to being transported to site for asphalt production.

All asphalt mixtures were prepared in a laboratory mixer at 160 °C, and volumetric and performance specimens were compacted at 142 °C, as detailed in the Australian standards for asphalt mixtures produced in the laboratory with C170 bitumen. As stated above, additional CRM binder content was required to produce equivalent Marshall air voids contents with increasing CR contents and for short-blending processes, due to the associated increases in binder viscosity associated with CRM, and the morphology and state of the rubber particles in the binder, due to reduced CR dissolution effects during short-duration blending [65].

### 3.2. Methods

Asphalt specimens were prepared, conditioned, and tested according to the various Australian and international standard test methods (Table 4). Three replicates were produced and tested for each mixture designation and each test method. For wheel tracking, the cumulative rut depth was recorded continuously, as required by the standard test method. For the moisture resistance testing, the unconditioned and conditioned tensile strength was measured for triplicate samples, providing a single TSR value for each mixture designation, as required by the standard test method.

## 4. Results and Discussion

### 4.1. Stiffness

**Figure 2:**
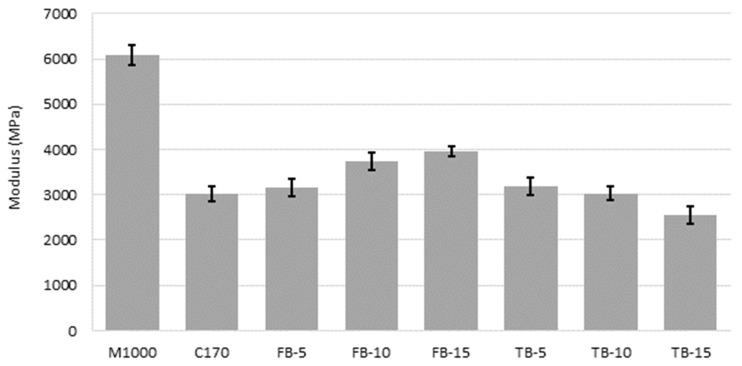
Average resilient modulus values.

The mixture stiffness results are shown in Table 5. The average values are compared in Figure 2. The error bars represent the average plus and minus the standard deviation for the triplicate specimen results. It is clear that M1000 was associated with the highest stiffness, and this was statistically significant when compared to all mixtures (*p*-values all <0.01) and reflects the plastomeric nature of the acid-modified multigrade binder. When compared to mixtures containing unmodified C170 binder, the CRM mixture stiffnesses were more comparable.

**Figure 3:**
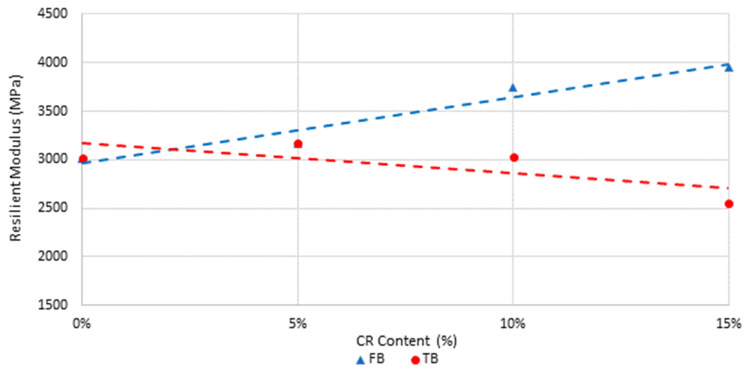
Effect of CR content and digestion time on resilient modulus.

The average stiffness increased with increasing CR content for the short-blend mixtures but decreased with increasing CR content for the long-blend mixtures, as shown in Figure 2 and highlighted in Figure 3. The differences were significant for FB-10 and FB-15 (*p*-values both 0.01) with the CRM mixture stiffness greater than the C170 mixture stiffness, as well as for TB-15 (*p*-value 0.04) but with the CRM mixture stiffness lower than the C170 mixture stiffness. This reflects the higher degree of dissolution achieved with the longer digestion time, resulting in a softening of the binder by the rubber compared to the short blending, where more rubber remains in a semi-solid state. Here, the state and morphology of the CR in the short blends from these reduced dissolution effects results in an increase in maltene fraction uptake of the base C170 binder from the diffusion induced swelling processes during reaction, and therefore an overall stiffening effect on the liquid phase of the CRM binder and the resulting asphalt mixture. Comparatively, the long-blend methods result in the increased dissolution and dispersion of the rubber polymer components and re-releases a portion of the maltene fraction into the binder phase, resulting in a reduction in the CRM binder and mixture stiffness [75]. This implies that the blending time of CRM binders can be used as a factor to adjust the stiffness of the resulting asphalt mixture, depending on the asphalt mixture application. The inverse is also true and shows how critical it is to control blending processes, temperatures, and durations at the asphalt mixture production plant to ensure that the intended mixture stiffness is achieved.

### 4.2. Deformation Resistance

**Figure 4:**
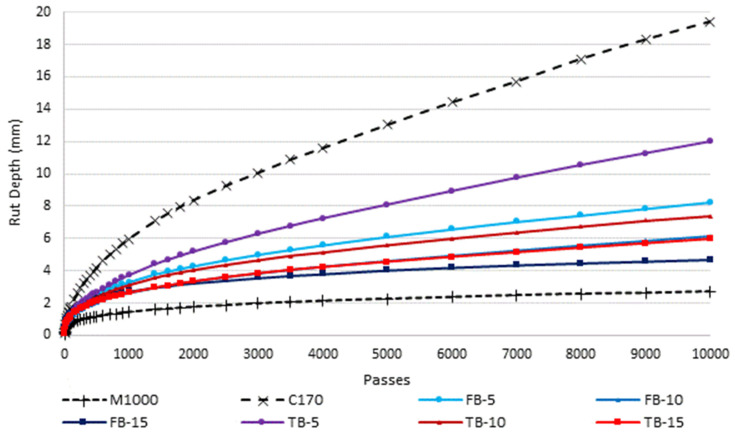
Wheel track deformation results.

**Figure 5:**
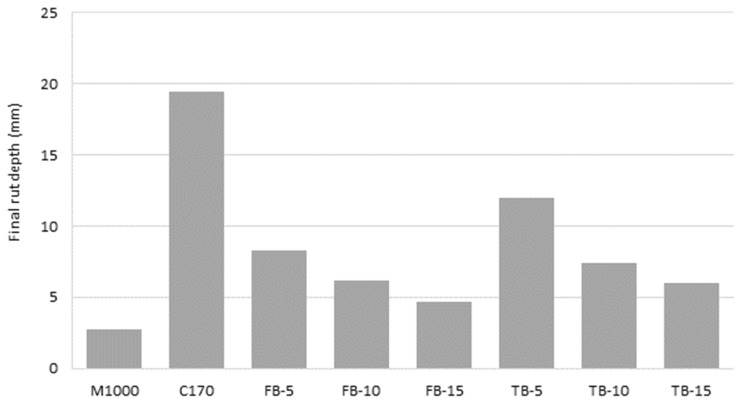
Terminal (10,000 passes) rut depth.

The wheel tracking results are shown graphically in Figure 4 and the terminal (10,000 passes) rut depths are displayed in Figure 5. The significantly greater rut resistance associated with the M1000 binder is clear, with a terminal rut depth of just 2.7 mm, which is very low for an asphalt mixture intended for local road surfacing use, being more comparable with heavy duty applications [69]. In contrast, the unmodified C170 was associated with the lowest rut resistance, with a terminal rut depth of 19.4 mm. All the CRM mixtures were associated with increased rut resistance, compared to C170, and the differences were significant in all cases (*p*-values 0.04 to less than 0.01). However, the CRM mixture deformation resistance was not as high as for the M1000, and those differences were also significant (*p*-values all >0.01). For both the short- and long-duration blended CR binders, the rut resistance increased as the CR content increased. For the short-blend products, the rut depths reduced from 8.2 mm to 4.7 mm, as the CR content increased from 5% to 15%. Similarly, for the long-blended CRM binders, the rut depths reduced from 12.0 mm to 6.0 mm for the same 5% to 15% CR content. Although the long-blended products were associated with consistently lower terminal rut depth than the equivalent short-blend products, the difference was not statistically significant on a paired basis (*p*-value 0.13). That is, the CR blending time did not significantly affect the resulting deformation resistance of the asphalt mixtures. The improved deformation resistance effects are consistent with other studies using low dose wet-blended CRM binders in dense-grade asphalt, where performance can be considerably improved proportional to the incorporated CR contents [85,86,87,88,89,90]. Similarly, other terminal blend or more fully digested CRM products are generally not as effective as short-blend products but still provide improved performance relative to their respective base binders [91,92].

The trend in long-blend rut resistance is opposite to the trend in stiffness. For the long-blended CRM binders, the mixture stiffness decreased with increasing CR content, whereas for deformation resistance, the performance increased with increasing CR content. This highlights that stiffness is not necessarily a good predictor of other performance properties, because stiffness is not necessarily directly related to ductility and brittleness, particularly across different types of materials, and in this case for differently modified binders. The Jnr_3.2_ (kPa^−1^) values from the multiple stress creep recovery test (Table 2) at the equivalent test temperature of 60 °C had a high degree of correlation with the tested rut depths (R^2^ = 0.926), indicating that modification effects from the CR polymer components provided improved deformation properties by improving non-recoverable creep compliance and binder elasticity. Similar trends are seen in elastomeric polymer-modified binders and mixtures, where a significant decrease in mixture stiffness results from the elastically modified mixture, but the increase in the binder resistance to flow, particularly at elevated temperatures, also significantly increases the mixture resistance to deformation [69].

### 4.3. Fatigue Resistance

**Figure 6:**
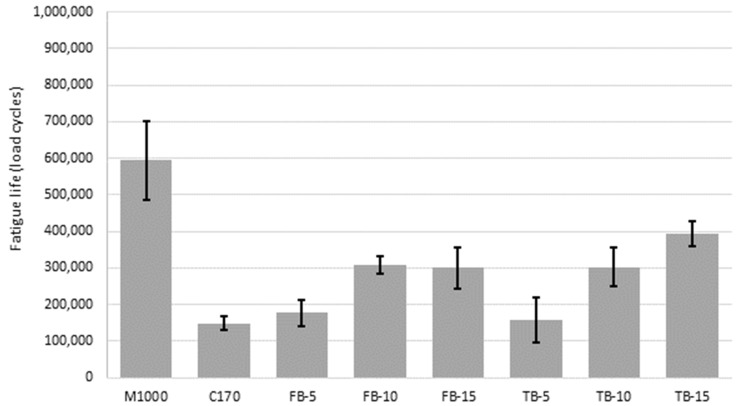
Average fatigue values.

The fatigue results, expressed as load cycles to the failure condition, are detailed in Table 6, for the triplicate specimens. The average fatigue lives for each mixture are compared in Figure 6, with error bars that represent the average value plus/minus the standard deviation for the triplicate results. The M1000 mixtures had the highest fatigue life. This again shows that the high stiffness associated with this binder does not necessarily result in a low fatigue life, meaning that stiffness is not necessarily an indicator of brittleness, and this has been similarly demonstrated by others [93]. In contrast, the unmodified C170 binder was associated with the lowest fatigue resistance. However, this was only 6% and 19% lower than for the long and short-blended CRM binders at 5% CR content, respectively. Furthermore, these small increases in fatigue life for 5% CR content binders were not statistically significant (*p*-values 0.29 and 0.82, for the short and long-blends, respectively). With increased CR content, the fatigue lives increased significantly (*p*-values 0.01 and below for all) and on an average-paired basis, the difference in the two blending durations was not significant (*p*-value 0.59). This is consistent with the deformation resistance results, where the 10% and 15% dosages of CR were associated with significant performance improvements, but the improvement was not as significant as for the M1000 binder and was not significantly different for the two blending durations. That is, the binder content had a significant effect on the performance properties, but the blending time did not.

These findings are consistent with other studies on short (field) and long (terminal) duration blending approaches, where fatigue performance generally increases with increasing CR contents [87,94,95,96,97]. This can be attributed to the increase in binder volumes and associated binder films, which combined with the release of the dissolved polymeric compounds and the remaining CR particles themselves, can improve binder cohesion as dispersed throughout the asphalt matrix.

### 4.4. Fracture Resistance

**Figure 7:**
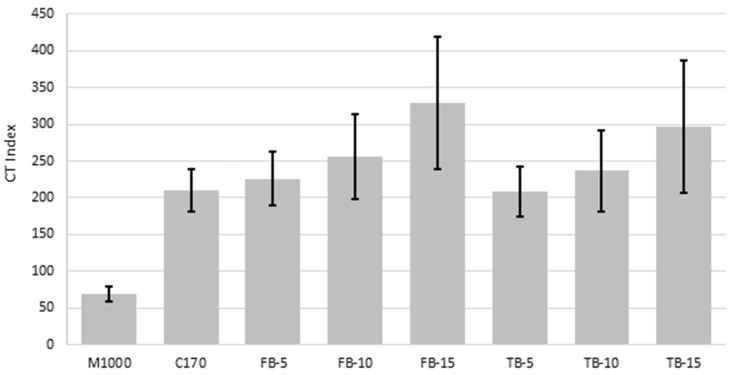
Average CT Index values.

The fracture resistance results, expressed as the CT index, are presented in Table 7. The average CT index for each mixture is compared in Figure 7, with error bars that represent the average value plus/minus the standard deviation for the triplicate results. In contrast to all other properties, including intermediate temperature fatigue resistance, the M1000 was associated with the lowest low temperature fracture resistance, with an average CT Index of just 69, compared to 209 for the unmodified C170 binder. This indicates that although M1000 binder is associated with good deformation and fatigue resistance, it is prone to low-temperature brittle fracture. This is consistent with other research that has found that M1000 binder is particularly prone to severe top-down cracking, particularly in non-loaded areas of airport pavements [83].

The average CT Index values followed a similar trend to the other performance-related properties in that the 5% to 15% CR contents were associated with increases in the CT Index, and that the improvement increased with increasing CR content. However, the higher variability associated with the CT Index test meant that these average improvements were not statistically significant (*p*-values 0.09 to 0.97). Interestingly, and in contrast to the previously considered properties, the blending duration was significant, with an average-paired *p*-value of 0.04, with the short-blends associated with better fracture resistance than the long-blends, which is consistent with more semi-solid CR remaining in the binder, resulting in a greater degree of improvement, similar to the stiffness results.

### 4.5. Moisture Resistance

**Figure 8:**
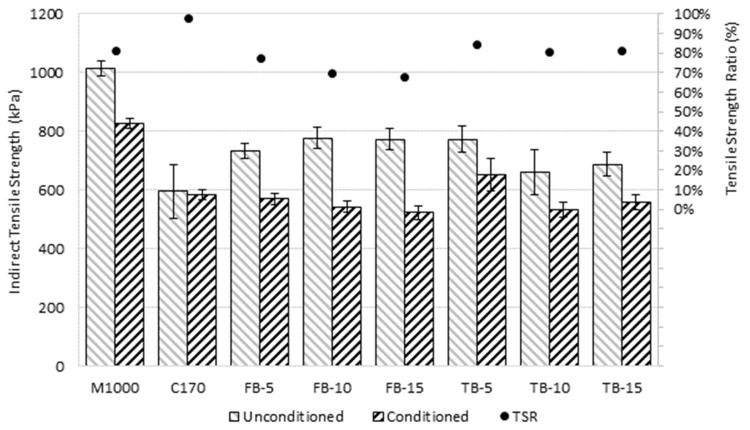
Average conditioned and unconditioned tensile strengths and TSR values.

The moisture damage resistance results, expressed as the indirect tensile strength for the conditioned and unconditioned specimens, are presented in Table 8. The average indirect tensile strength values and the calculated TSR value for each mixture are compared in Figure 8, with error bars that represent the average value plus/minus the standard deviation for the triplicate results. The M1000 binder was associated with the highest indirect tensile strength (ITS) in both the unconditioned and the conditioned states, at an average of 1015 kPa and 825 kPa, respectively. Compared to the unmodified C170, the CR-modified binders were generally associated with a slight increase in average unconditioned ITS and a slight reduction in the conditioned ITS. This resulted in a reduction in the TSR values for the CRM mixtures, particularly the short-blended products with increasing CR contents. In fact, the short-blend CRM mixtures had TSR values of 68% to 78%, which are all below the 80% generally recommended as being indicative of stripping resistance mixtures [69].

In contrast, the long-duration blends were associated with TSR values of 81% to 85%, which are all above the 80% threshold. This resulted in a significant difference in the TSR values for the two blending durations on a paired basis (*p*-value 0.03) even though the ITS values were not significantly different on an average-paired basis, with *p*-values of 0.20 (unconditioned) and 0.32 (conditioned). This indicates that the long-blend products may be better for stripping resistance than the short-blend products, and this may reflect the greater degree of CR digestion resulting in a more homogenous product that is better able to adhere and bond to the aggregate particles.

The reduction in TSR values for the CRM blends may be attributed to reduced adhesive properties, resulting from the reduction in aromatics due to absorption of these components into the rubber crumb during blending [98]. As no extender oils were used in the manufacture of the CRM binders, lower initial aromatic contents of the base binder and greater relative absorption through increasing CR contents may have significantly reduced the concentration of light oil fractions post-reaction [99]. This is consistent with other studies examining the effects of CRM binder adhesive properties, which determined that increasing CR contents resulted in a reduction in adhesion in asphalt mixtures, due in part to the inability of the undissolved CR particles to bond to the aggregates [100,101,102,103,104]. Similarly, the increase in the viscosity of the CRM binder may inhibit mixture workability and absorption of the binder film into the aggregate surface, effectively reducing mixture adhesion properties through the mechanisms above, particularly after moisture conditioning [105]. Limiting CR contents below 10% may help to mitigate these issues, without additional binder modification or use of extender oils [106]. Conversely, the relative improvement for the long-duration blends is consistent with CR particle degradation and dissolution effects from extended blending durations, where the comparative increase in aromatics in the liquid binder phase, relative to short-blends, benefits in improved binder-aggregate adhesion. Given the high variability associated with the IDT values and only one TSR value per mixture type, further work is recommended if short-blend CRM binders are considered for routine use.

## 5. Conclusions

Based on a laboratory comparison of common performance-related mechanical properties in an otherwise nominally identical aggregate skeleton, it was concluded that CRM binders generally improved the performance of the asphalt mixtures, compared to unmodified C170 bitumen, but the degree of modification was not as substantial as for the acid-modified multigrade (M1000) binder. As shown in Table 9, the effect of blending duration was only sometimes significant, but the trend in the results were generally similar for the short-blend and long-blend durations. This indicates that the decision to use field-blended or terminal-blended products should be made based on economics and logistics, and not on field performance. Furthermore, the effect of CRM generally increased as the CR content increased, with 5% CR content often being insignificant but 10% and 15% CR content being significantly different to the unmodified C170 binder. Consequently, 10% is concluded as being the optimal CR content for load road surface applications, given the increase in cost associated with higher CR contents.

Overall, it was concluded that low dosage CRM asphalt mixtures were not associated with any significant detrimental effects, regardless of the CR content or the blending duration. The only potential performance reduction was associated with stiffness and moisture resistance. Stiffness is generally not important for routine resurfacing of local roads, and the moisture damage resistance was associated with limited data that warrants further consideration in future research. However, the anti-ageing potential for these binders and mixtures needs to be compared in order to understand whether the increase cost in the binder results in an improved expected surface life, and whether the net effect is a better whole of life proposition. That research can now be progressed in the future, without concern for the common performance-related mechanical properties, for an improved whole life-cycle cost of local road pavement surfacing. Although this study found positive results, it is inherently limited by considering only one asphalt mixture type and one CR supply. Further testing of different mixture types and CR sources would be required to generalize these conclusions.

## Figures and Tables

**Table 1:** Asphalt mixture binder contents.

Sample Description	Modified Binder Content (by Mass)
Control with M1000 bitumen	5.9%
Control with C170 bitumen	5.9%
Short-blended 5% crumb rubber	6.1%
Short-blended 10% crumb rubber	6.5%
Short-blended 15% crumb rubber	7.0%
Long-blended 5% crumb rubber	6.0%
Long-blended 10% crumb rubber	6.4%
Long-blended 15% crumb rubber	6.8%

**Table 2:** Binder properties.

Property	Torsional Recovery	Penetration	Softening Point	Viscosity at 60 °C	Segregation	J_nr3.2_ at 60 °C (MSCR) (RTFO)
Units	(%)	(Units)	(°C)	(Pa.s)	(%)	(kPa^−1^)
Method	AGPT/T122	AGPT/T108	AGPT/T131	AGPT/T131	AGPT/T108	AASHTO T350
M1000	9	40.5	56.7	1354	N/A	0.126
C170	4	65.7	48.3	203	N/A	2.101
FB-5	14	60.7	51.2	474	6	1.268
FB-10	21	54.7	57.8	947	8	0.568
FB-15	41	50.4	62.4	1712	12	0.285
TB-5	14	65.2	50.7	461	5	1.192
TB-10	25	63.8	54.0	691	18	0.749
TB-15	28	64.5	56.5	1116	18	0.468

N/A = test method not applicable to this type of binder.

**Table 3:** Sample designations and characteristics.

Designation	CR Content	CR Blending Duration
M1000	Not applicable	Not applicable
C170	Not applicable	Not applicable
FB-5	5%	Short
FB-10	10%	Short
FB-15	15%	Short
TB-5	5%	Long
TB-10	10%	Long
TB-15	15%	Long

CR contents are by mass of the unmodified bitumen.

**Table 4:** Asphalt test methods.

Property	Method	Description
Resilient modulus	AS/NZS 2891.13.1	Indirect tensile modulus at 25 °C, an indicator of relative material stiffness
Wheel tracking	AGPT/T231	Cooper’s wheel tracker to 10,000 passes at 60 °C, an indicator of relative deformation resistance at high in-service temperatures
Fatigue life	AGPT/T274	The number of four-point bending cycles at 20 °C and 200 µɛ sinusoidal until the modulus is reduced to 50% of the initial modulus, an indicator of relative fracture resistance at intermediate in-service temperatures
IDEAL-CT	ASTM D8225	An energy-based index calculated from monotonic loading at 50 mm/min and 25 °C, an indicator of intermediate temperature crack resistance
Tensile strength ratio	AGPT/T232	Modified Lottman test, as the ratio between the average unconditioned an indicator of moisture damage (stripping) resistance

**Table 5:** Resilient modulus results.

Mixture	Resilient Modulus (MPa)		
	Specimen 1	Specimen 2	Specimen 3
M1000	5836	6142	6262
C170	2850	3010	3180
FB-5	2950	3240	3310
FB-10	3692	3584	3947
FB-15	4070	3880	3920
TB-5	3015	3130	3384
TB-10	2883	3194	3011
TB-15	2340	2564	2742

**Table 6:** Four-point bending fatigue life results.

Mixture	Fatigue Life (Cycles to Failure)		
	Specimen 1	Specimen 2	Specimen 3
M1000	518,210	669,240	593,725
C170	152,090	165,630	127,800
FB-5	215,670	173,390	143,730
FB-10	306,340	283,420	332,130
FB-15	239,910	304,790	354,510
TB-5	87,570	200,060	184,220
TB-10	357,480	297,500	251,820
TB-15	412,270	352,560	414,750

**Table 7:** IDEAL CT results.

Mixture	CT Index		
	Specimen 1	Specimen 2	Specimen 3
M1000	65.4	60.3	79.9
C170	239.8	182.6	205.5
FB-5	184.3	248.9	263.5
FB-10	245.6	317.3	203.8
FB-15	272.7	432.2	281.0
TB-5	177.1	244.2	203.1
TB-10	297.4	188.7	224.6
TB-15	243.9	245.8	401.4

**Table 8:** Indirect tensile strength results (kPa).

Mixture	CT Index					
	Unconditioned Specimens			Conditioned Specimens		
	Specimen 1	Specimen 2	Specimen 3	Specimen 4	Specimen 5	Specimen 6
M1000	1025	985	1034	816	846	816
C170	490	627	666	597	567	588
FB-5	732	707	761	592	555	561
FB-10	802	793	735	536	565	527
FB-15	812	766	739	531	497	542
TB-5	752	741	823	607	637	715
TB-10	607	627	746	509	558	529
TB-15	697	720	642	540	588	550

**Table 9:** Summary of effects.

Property	Short Duration (FB)	Long Duration (TB)
Stiffness	Improvement, increasing with increasing CR content	Comparable, but decreasing with increasing CR content
Deformation resistance	Significant improvement, increasing with increasing CR content	Significant improvement, increasing with increasing CR content
Fatigue resistance	Significant improvement at 10% and 15%, increasing with increasing CR content	Significant improvement at 10% and 15%, increasing with increasing CR content
Fracture resistance	Insignificant but consistent improvement, increasing with increasing CR content	Insignificant but consistent improvement, increasing with increasing CR content
Moisture resistance	Significant reduction	Insignificant average reduction

## Data Availability

The original contributions presented in this study are included in the article. Further inquiries can be directed to the corresponding author.
